# Assessing the performance of national sentinel food lists at subnational levels in six countries

**DOI:** 10.1017/S1368980023002823

**Published:** 2023-12-15

**Authors:** Chris Vogliano, Veronica Varela, Monica Woldt, Silvia Alayon, Laura S Hackl, Gina Kennedy, Sarah H Pedersen, Erin M Milner, Jennifer Yourkavitch

**Affiliations:** 1 USAID Advancing Nutrition, Arlington, VA 22202, USA; 2 Helen Keller International, New York, USA; 3 John Snow, Inc., Arlington, VA, USA; 4 Save the Children, Washington, DC, USA; 5 Global Alliance for Improved Nutrition, Washington, DC, USA; 6 Credence/USAID Global Health Technical Professionals, Washington, DC, USA; 7 Public Health Institute/USAID Sustaining Technical and Analytical Resources, Washington, DC, USA; 8 Results for Development, Washington, DC, USA

**Keywords:** Diet quality, Diet diversity, Food lists, Diet measurement, Low- and middle-income countries

## Abstract

**Objective:**

To assess how well national sentinel lists of the most frequently consumed foods in each food group capture data at subnational levels to measure minimum diet diversity (MDD).

**Design:**

We analysed data from seven surveys with 24-h open dietary recalls to evaluate: (1) the percentage of reported foods that were included in each sentinel food list; (2) whether these lists captured consumption of some food groups better than others and (3) differences between estimates of dietary diversity calculated from all food items mentioned in the open 24-h recall *v*. only food items included in the sentinel lists.

**Setting:**

Seven subnational areas: Bangladesh (2), Benin, Colombia, Kenya, Malawi and Nepal.

**Participants:**

8094 women 15–49 years; 4588 children 6–23 months.

**Results:**

National sentinel food lists captured most foods reportedly consumed by women (84 %) and children (86 %). Food groups with the highest variability were ‘other fruits’ and ‘other vegetables.’ MDD calculated from the sentinel list was, on average, 6·5 (women) and 4·1 (children) percentage points lower than when calculated from open 24-h recalls, with a statistically significant difference in most subnational areas.

**Conclusion:**

National sentinel food lists can provide reliable data at subnational levels for most food groups, with some variability by country and sub-region. Assessing the accuracy of national sentinel food lists, especially for fruits and vegetables, before using them at the subnational level could avoid potentially underestimating dietary diversity and provide more accurate local information for programmes, policy and research.

High quality diets are essential for preventing malnutrition and non-communicable diseases. In many low- and middle-income countries (LMIC), diets lack diversity and sustainable access to nutritious foods can be limited^(1)^. Concurrently, many LMIC are experiencing a shift in dietary intakes characterised by a transition away from minimally processed, fresh whole foods to a diet pattern that includes more pre-packaged foods, even among households experiencing food insecurity^(2–4)^. Despite the strong relationship between poor quality diets and adverse health outcomes, limited data exist on dietary intake, particularly in LMIC undergoing a rapid diet transition. While some large-scale population-based household surveys, like Demographic and Health Surveys, capture dietary intake in LMIC at national or regional levels approximately every 5 years, these have historically relied on methods that included open response options with non-standardised methods for classifying foods into food groups^(5,6)^.

Few simple, low-cost methods exist to routinely collect dietary intake data to assess trends in food consumption^(7–9)^. Common methods to assess dietary intake involve quantitative assessments such as 24-h open dietary recalls or weighed food records^(10–12)^. These dietary assessment techniques can provide detailed data on food consumption, but data collection and analysis are complex, requiring well-trained data collectors, analysts, time and funding^(10,13,14)^.

One effort to overcome these challenges is the development of simple, low-burden and rapid diet quality questionnaires (DQQ) that use national-level sentinel food lists to collect data to monitor diet quality using standard indicators such as minimum dietary diversity for women (MDD-W) and children (MDD)^(15–17)^. Sentinel foods are defined as frequently consumed, context-specific foods within specific food groups that capture a large proportion of people consuming anything in that food group^(18)^. Enumerators use these questionnaires by reading a list of foods in each food group to respondents and noting the food groups the respondent indicates to have consumed in the past 24 h. The DQQ were developed through expert consultations^(19)^ to identify culturally appropriate, nationally representative and country-specific sentinel foods across twenty-nine unique food groups (see online supplementary material, Supplemental Material I). DQQ are available for adults in over 100 countries and for infants and young children aged 6–23 months in ninety-two countries^(20–22)^. The DQQ were validated with nationally representative 24-h dietary intake data from Brazil (2008–2009) and the USA (2009–2014). The validation showed that 1–7 sentinel foods per food group captured, on average, 96–97 % of people who consumed any foods in a respective food group^(20)^.

The aim of the national sentinel food lists is to capture at least 90% of people who consumed any item in each food group^(15)^. However, it is unclear if these lists accurately capture food group consumption at subnational levels. The purpose of this study is to evaluate if the national sentinel food lists capture commonly consumed foods at subnational levels; if the lists capture some food groups better than others and if the differences between what the lists do or do not capture affect dietary diversity indicators. These results will inform the use of national sentinel food lists for planning and monitoring nutrition programmes in subnational areas.

## Methods

### Identifying datasets

We performed a secondary analysis of dietary intake data. We identified eligible datasets for this analysis by searching the following four databases: (1) FAO/ WHO Global Individual Food consumption data Tool database of publicly available 24-h open recall data^(23)^; (2) Global Dietary Database^(24)^, (3) Harvard Dataverse project^(25)^, (4) PubMed^(26)^ and USAID data (personal communication; Sarah Pedersen, USAID Bureau for Resilience and Food Security nutrition advisor, 1 October 2022). We then followed up individually with owners of eligible datasets.

We established these inclusion criteria for datasets:

- Quantitative or qualitative 24-h open dietary recall data or weighed food record data for women aged 15–49 years, AND/OR infants and young children 6–23 months of age in LMIC; AND
- Data were representative at a subnational administrative level (community, subdistrict, district, province, region or a composite of two or more subnational areas (e.g. a programme area); AND
- Data were collected within the last 10 years (January 2011–July 2022); AND
- National sentinel food lists for women aged 15–49 years and children aged 6–23 months for the country had been finalised at the time of the analysis (July 2022).

**Fig. 1:**
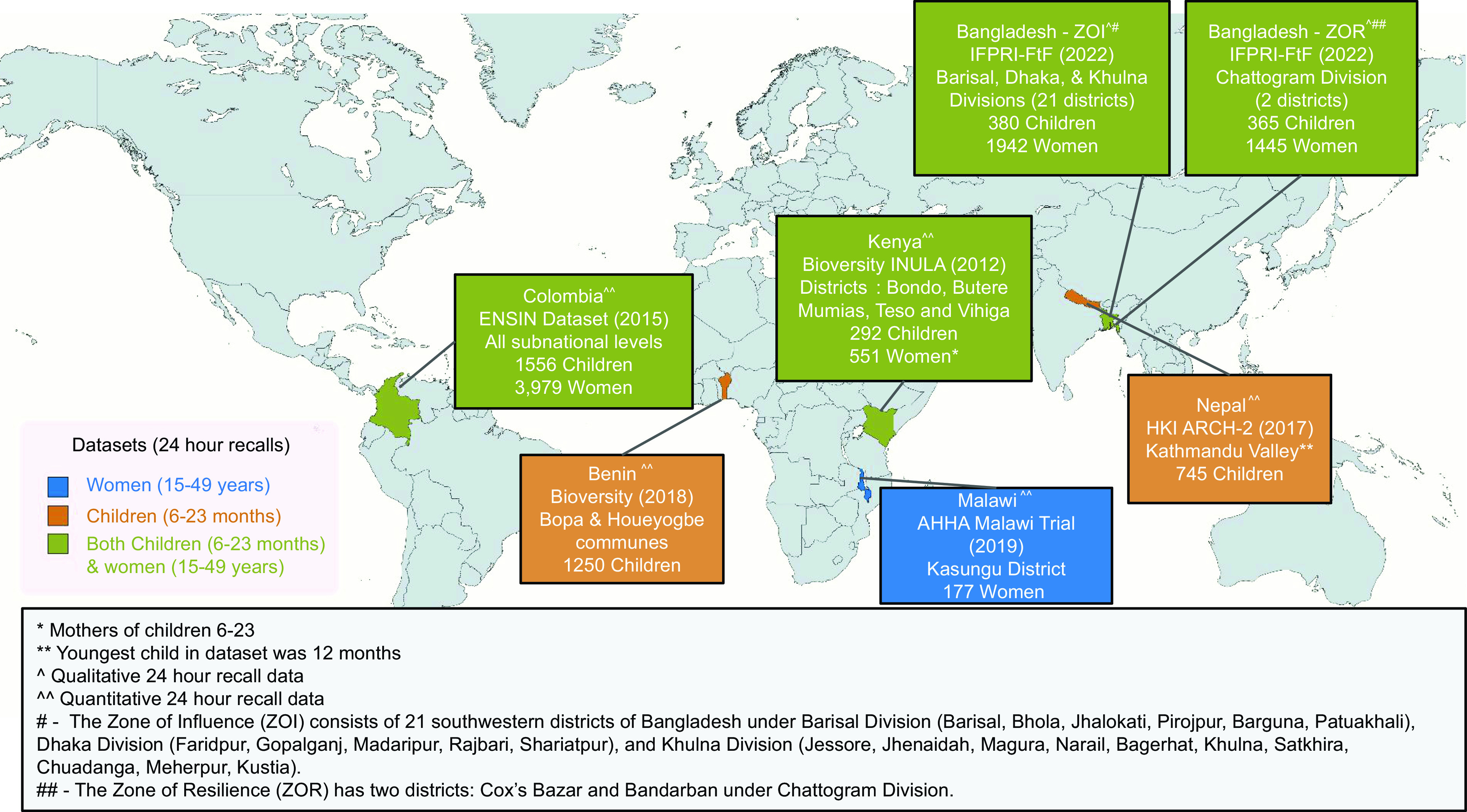
Map of 24-h dietary recall datasets used in this analysis, including demographics, sub-national regions and sample sizes

We obtained datasets with 24-h open dietary recalls from 8094 women aged 15–49 years and 4588 children aged 6–23 months in seven subnational areas of six countries: Bangladesh (2, identified as the zone of influence (ZOI) and zone of resilience (ZOR), which are operational areas for the US Government’s Feed the Future initiative and US Agency for International Development’s Bureau for Resilience and Food Security, respectively); Benin; Colombia; Kenya; Malawi and Nepal (Fig. 1). For all countries, we used the sub-regions identified in the datasets, except for Colombia, where six sub-regions were identified. Due to study resource constraints, we randomly selected three sub-regions there and combined them to achieve a manageable sample size.

### Data coding

For each country and population (i.e. women or infants and young children), we downloaded the sentinel food list, organised by food group, from the dietquality.org website and used it as the reference. We obtained the lists of food groups used to calculate MDD-W and MDD from the most recent global guidelines^(16,17)^. Two staff independently coded the foods in each dataset and resolved conflicts through discussion between them or with the full study team. Each food in the dataset was coded ‘yes/no’ to reflect whether it was included in the sentinel food list for that country (sentinel food status). We then assigned each food to the appropriate DQQ food group(s) and MDD-W or MDD food group(s) (see online supplementary material, Supplemental Material 1). We excluded food items from the analysis if they were not included in either the DQQ or MDD-W/MDD food groups (e.g. oils and spices). We referenced the FAO’s *Updated Guide on Minimum Dietary Diversity for Women* ^(16)^ for questions surrounding classification of food items into food groups. For example, a food item described as ‘peas, green, eaten as fresh pod’ was classified as ‘other vegetables’, while a food item described as ‘pea, mature seed’ was classified under ‘pulses’. Fried foods were included in the group that described the food prior to frying, unless they were ultra-processed foods, such as chips and crisps^(16)^, and processed meats were classified as flesh foods.

Most datasets for women 15–49 years and for children 6–23 months included the names of foods reportedly consumed and the quantity (in g) of each food consumed for each respondent. Following global guidance^(16)^, this analysis included only foods that women reportedly consumed in quantities of 15 g or more and datasets from Bangladesh (the only qualitative datasets) included only the names of foods that women consumed in quantities of 15 g or more. For children, this analysis included all foods regardless of the quantity consumed, following global guidance^(17)^. For mixed dishes or dishes with two or more primary ingredients, we used the total quantity of the dish consumed (g) to classify the components of the dish into food groups. We assigned each main ingredient food item (generally two or three ingredients) a sentinel food status, DQQ food group and MDD-W/MDD food group according to this procedure:

- Mixed dishes consumed in quantities of 15–30 g: categorised only the primary food item in the dish into a food group
- Mixed dishes consumed in quantities of 31–45 g: categorised only the two most abundant food items contained in the dish into food groups
- Mixed dishes consumed in quantities greater than 45 g: categorised the three most relevant food items contained in the dish into food groups.

For example, in a dish containing more than one food item, described as an arepa with cheese eaten in a quantity of 150 g, we assigned each food item in the dish (arepa and cheese) a sentinel status, DQQ food group and MDD-W/MDD food group code separately. The arepa was classified according to the FAO classification, ‘grains, white roots and tubers, and plantains’, while the cheese was classified as ‘milk and milk products’.

### Data analysis

Data were analysed using R (version 4.1.0) statistical software. We calculated descriptive statistics for each research question (Table 1) and used McNemar’s test to identify significant differences in the proportion of respondents who consumed each food group, based on the national sentinel food lists and the MDD-W/MDD calculation guidelines. We defined a ‘qualifying food’ as any food we could categorise into an MDD-W/MDD food group based on the indicator guidelines^(16,17)^. We defined a ‘sentinel qualifying food’ as a food that appears on the national sentinel food list.

**Table 1:** Research questions, measures and analytic method

Research Questions	Measures	Analytic method
1. Are any of the foods commonly consumed at the subnational level not included in the national sentinel food lists?	Percentage of specific food items, by food group, named in the 24-h open dietary recall, which meet the criteria to include them in an MDD-W food group* or an MDD food group^†^, which did not appear on the respective national sentinel food list	Descriptive statistical analyses, by country
2. Do the national sentinel food lists capture consumption of some food groups more accurately than others for women 15–49 years and/or children 6–23 months?	Percentage point difference between respondents who consumed any qualifying food in the MDD-W or MDD food group and those who consumed at least one sentinel food, by MDD-W/MDD food groups	Descriptive analyses and McNemar’s test for paired proportions, by country
3. In cases where differences in food group consumption exist, do the differences affect the estimates of MDD-W or MDD?	Percentage of children 6–23 months of age who consumed foods and beverages from at least five out of eight defined food groups during the previous day, comparing two methods: 1. Counting food groups only if the child consumed one of the sentinel foods 2. Counting food groups if the child consumed any of the MDD qualifying foods Percentage of women 15–49 years who consumed foods and beverages from at least five of ten defined food groups during the previous day, comparing two methods: 1. Counting food groups only if the woman consumed one of the sentinel foods 2. Counting food groups if the woman consumed any of the MDD-W qualifying foods	MDD-W or MDD estimates and McNemar’s test for paired proportions, by country

MDD, minimum diet diversity.

* As noted in reference 22.

† As noted in reference 21.

## Results

### 1. Are any of the foods commonly consumed at the subnational level not captured by the national sentinel food lists?

**Fig. 2:**
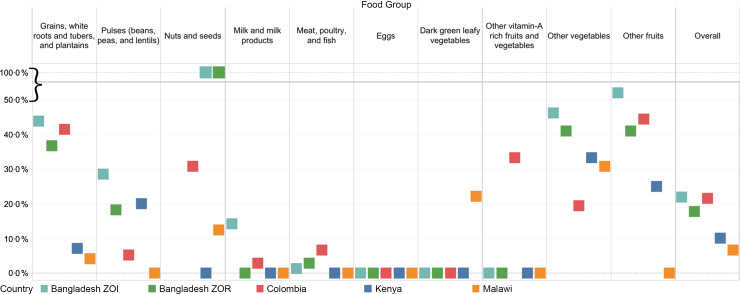
Percentage of foods named in the 24-h open dietary recalls that were not included in the national sentinel food list for the ten MDD-W food groups

The national sentinel food lists captured the majority of foods reported in the dietary recalls for women (average: 84 %; range: 78 % in the Bangladesh ZOI to 93 % in Malawi; Fig. 2; see online supplementary material, Supplemental Material 2.1). Across all countries, the national sentinel food lists captured a smaller proportion of foods consumed in the ‘other fruits’ and ‘other vegetables’ food groups compared with the other food groups. For the food group ‘other fruits’^(16)^, the national sentinel food list did not capture 52 % of foods in the Bangladesh ZOI, 41 % in the Bangladesh ZOR, 25 % in Kenya and 45 % in Colombia. In the Bangladesh ZOI, 46 % of foods categorised as ‘other vegetables’ were not included in the national sentinel list; this compares to 41 % in the Bangladesh ZOR, 33 % in Kenya, 31 % in Malawi and 19 % in Colombia. The food group ‘grains, white roots and tubers, and plantains’ had higher proportions of foods not captured by the national sentinel food lists in three of the five countries (44 % in the Bangladesh ZOI; 37 % in the Bangladesh ZOR and 41 % in Colombia). Notably, the sentinel list did not include foods in the ‘nuts and seeds’ food group in either of the datasets from Bangladesh. We identified one food – onion – in the Bangladesh ZOR and ZOI not included on the sentinel list but reportedly consumed by more than 90 % of respondents.

**Fig. 3:**
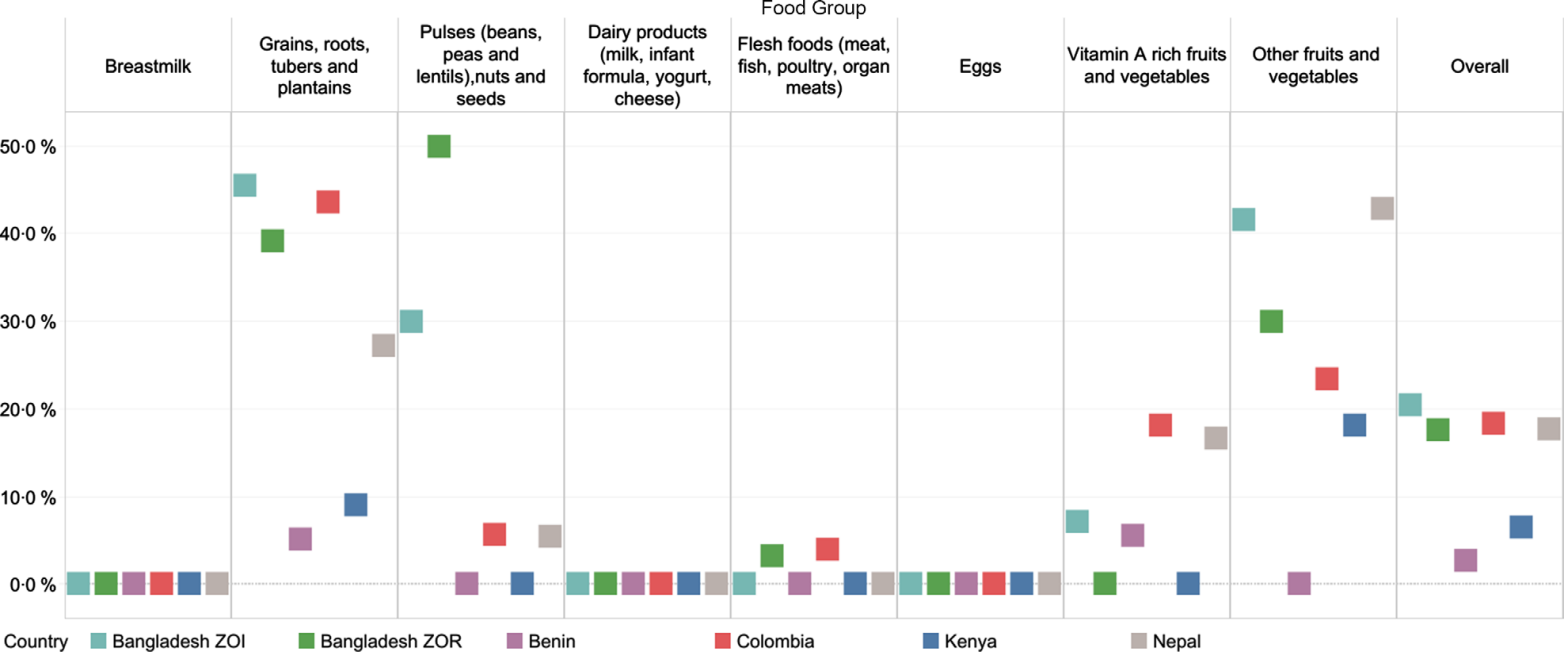
Percentage of foods named in the 24-h open dietary recalls that were not included in the national sentinel food list for the eight MDD food groups

Similarly, the national sentinel lists for children included most foods (average: 86 %; range: 80 % in the Bangladesh ZOI to 97 % in Benin) reported in the subnational dietary recalls (Fig. 3; see online supplementary material, Supplemental Material 2.2). Across all countries, the food groups ‘grains, roots, and tubers’ and ‘other fruits and vegetables’ had higher proportions of foods not captured by the national sentinel lists compared with other groups. In the Bangladesh ZOI, 45 % of foods categorised in the ‘grains, roots, and tubers’ food group were not included in the national sentinel list, as compared with 44 % in Colombia, 39 % in the Bangladesh ZOR, 27 % in Nepal, 9 % in Kenya and 5 % in Benin. In Nepal, 43 % of the foods categorised in the ‘other fruits and vegetables’ food group were not included; neither were 42 % in the Bangladesh ZOI, 30 % in the Bangladesh ZOR, 23 % in Colombia and 18 % in Kenya (see online supplementary material, Supplemental Materials 2.2 and 3.2).

### 2. Do the national sentinel food lists capture consumption of some food groups more accurately than others for women 15–49 years and/or children 6–23 months?

Across most countries, the sentinel food lists captured at least 90 % of women and children reported to have consumed foods from each food group. Two exceptions were the Bangladesh ZOI and ZOR, where the DQQ sentinel food list captured only 63 and 84% of women who reported consuming ‘other vegetables’, respectively, and 81% (ZOI) and 79% (ZOR) of children consuming ‘other fruits and vegetables’.

**Fig. 4:**
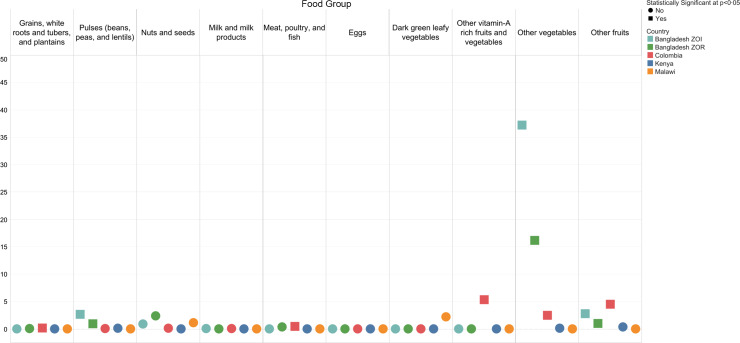
Percentage point difference between women who reported consuming any qualifying food in the MDD-W food groups and those who reported consuming at least one sentinel qualifying food*. *When interpreting the percentage point difference in the reported consumption of a food group, if there was a difference of zero, then all respondents who reported consuming any qualifying food in a certain food group also reported consuming at least one sentinel qualifying food in that food group. The reported consumption from a food group was not different when considering all qualifying foods compared with just sentinel qualifying foods. If there was a percentage point difference of 40, then the percentage of respondents who reported consuming foods from a food group was 40 points higher when considering all qualifying foods compared with considering just sentinel qualifying foods

We observed small differences between women’s reported consumption of food groups and inclusion in the sentinel lists (Fig. 4; see online supplementary material, Supplemental Material 2.3). Across datasets, there were no clear patterns regarding the magnitude of the difference for each food group. Notably, in the Bangladesh ZOI and ZOR there was a large statistically significant difference (37 and 16 percentage points, respectively; *P* < 0·05) between the reported consumption of the ‘other vegetables’ food group and what the sentinel list captured. We observed statistically significant differences in reported consumption for several food groups in the Bangladesh ZOI, Bangladesh ZOR and Colombia datasets. In the Bangladesh ZOI and ZOR, approximately 1 and 2% of women, respectively, reported consuming nuts and seeds; however, the sentinel list did not capture this.

**Fig. 5:**
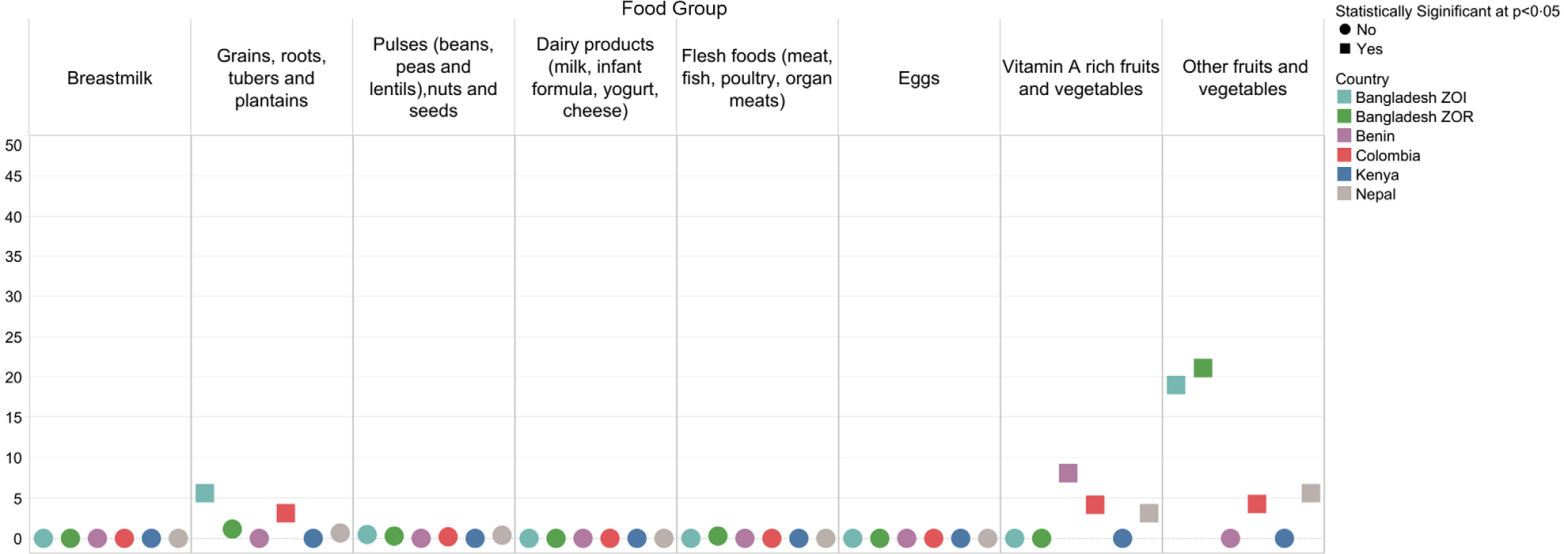
Percentage point difference between children who reported consuming any qualifying food in the MDD food groups and those who reported consuming at least one sentinel qualifying food*. *When interpreting the percentage point difference in the reported consumption of a food group, if there was a difference of zero, then all respondents who reported consuming any qualifying food in a certain food group also reported consuming at least one sentinel qualifying food in that food group. The reported consumption from a food group was not different when considering all qualifying foods compared with just sentinel qualifying foods. If there was a percentage point difference of 40, then the percentage of respondents who reported consuming foods from a food group was 40 points higher when considering all qualifying foods compared with considering just sentinel qualifying foods

Among children 6–23 months of age, there were differences between reported consumption of food groups and those captured by the sentinel lists; most differences were less than 10 percentage points (Fig. 5; see online supplementary material, Supplemental Material 2.4). In the Bangladesh ZOI and Colombia, there were significant differences in the reported consumption of the ‘grains, roots, and tubers’ food group (6 percentage points and 3 percentage points, respectively). There were significant differences in the reported consumption of vitamin A-rich fruits and vegetables in Benin (8 percentage points), Nepal (3 percentage points) and Colombia (4 percentage points). There were also significant differences in the reported consumption of the ‘other fruits and vegetables’ food group in the Bangladesh ZOR (21 percentage points), the Bangladesh ZOI (19 percentage points), Nepal (6 percentage points) and Colombia (4 percentage points).

### 3. In cases where differences in food group consumption exist, do the differences affect the estimates of MDD-W or MDD?

**Fig. 6:**
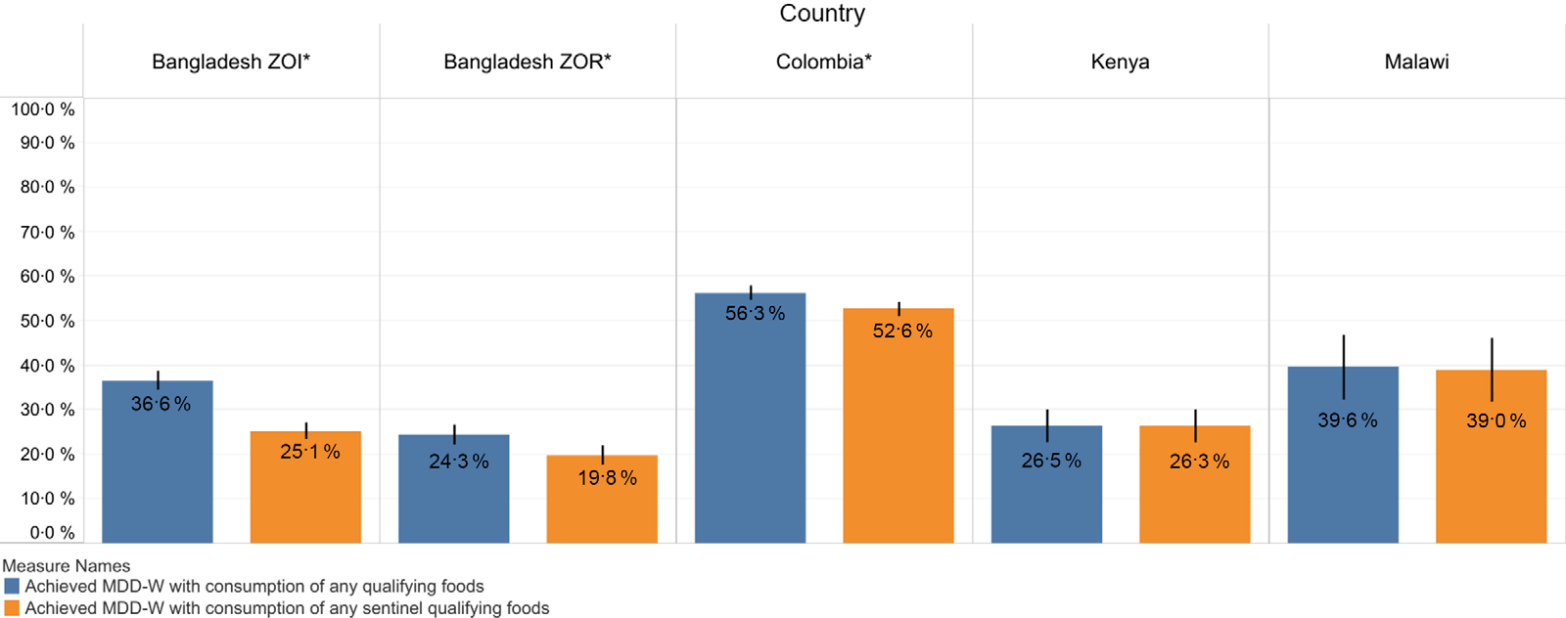
MDD-W among women 15–49 years old by reported consumption of any qualifying MDD-W foods and any sentinel qualifying foods. *Statistically significant at *P* < 0·05

In the Bangladesh ZOI, the Bangladesh ZOR and Colombia, there were significant differences between estimates of MDD-W based on reported consumption of any qualifying food (open recall) compared with reported consumption of any sentinel qualifying food using the sentinel food list (e.g. 37 % compared with 25 % in the Bangladesh ZOI). The ratio of the percentage of women who achieved the MDD-W with any sentinel qualifying food to the percentage who achieved the MDD-W with a qualifying food was 69%, indicating that the national sentinel list underestimated consumption by 31 % compared with the open recall. In the Bangladesh ZOR, 24 % of women achieved the MDD-W with any qualifying foods, compared with 20 % with sentinel qualifying foods (the sentinel list underestimated by 19 %), while the corresponding values in Colombia were 56·3 and 52·6 % (the sentinel list underestimated by 7 %; Fig. 6). We observed no significant differences in Kenya and Malawi, where there was less than one percentage point difference between the methods.

**Fig. 7:**
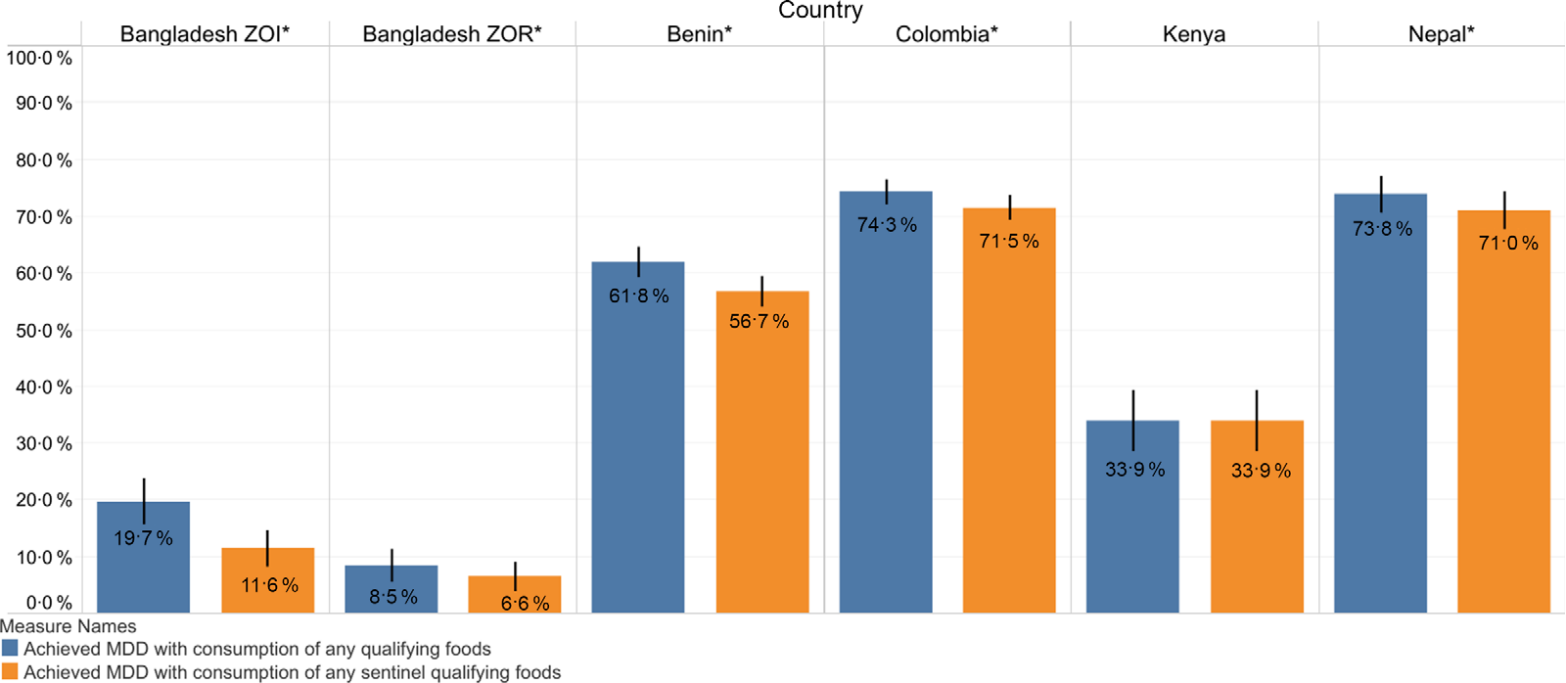
MDD among children 6–23 months by reported consumption of any qualifying MDD food and any sentinel qualifying foods. *Statistically significant at *P* < 0·05

For children 6–23 months of age, there were significant differences between MDD estimated from reported consumption of any qualifying MDD food and MDD estimated from consumption of any sentinel qualifying food (Fig. 7): Bangladesh ZOI (19·7 % *v*. 11·6 %, the sentinel list underestimated by 41 %); the Bangladesh ZOR (8·5 % *v*. 6·6 %, the sentinel list underestimated by 22 %); Benin (61·8 % *v*. 56·7 %, the sentinel list underestimated by 8 %); Colombia (74·3 % *v*. 71·5 %, the sentinel list underestimated by 4 %) and Nepal (73·8 % *v*. 71·0 %, the sentinel list underestimated by 4 %). We observed no significant differences in Kenya (see online supplementary material, Supplemental Material 2.6).

## Discussion

This study assessed whether national sentinel food lists capture foods consumed in subnational areas in Bangladesh, Benin, Colombia, Kenya, Malawi and Nepal; whether those lists capture consumption of foods in some food groups better than others and whether observed differences in food group consumption affect estimates of diet diversity indicators (MDD-W; MDD). We found that sentinel food lists, on average, captured 84 % of foods consumed at the subnational level for women and 86 % for children, with considerable variability among the regions studied. When comparing sentinel food lists to 24-h dietary recalls for women, Malawi’s list captured the most foods at the subnational level (93 %), and the Bangladesh ZOI captured the fewest (78 %). For children, Benin’s sentinel food list captured the most foods (97 %), while the Bangladesh ZOI captured the fewest (79 %).

The aim of sentinel food lists is to accurately capture over 90% of people who consumed at least one item in a food group^(15)^. The Bangladesh ZOI and ZOR had food groups with the largest differences between the percentage of people who reported consumption and foods captured by the sentinel food lists. For women, the food group with the largest difference was ‘other vegetables’; the sentinel list captured less than 90 % of women consuming that food group. There were also notable differences in the ‘other fruits’ food group. Similarly, the sentinel food list for Bangladesh captured less than 90 % of children consuming ‘other fruits and vegetables’ in the ZOI and ZOR with the ‘grains, roots, tubers, and plantains’ food group also showing notable differences.

We found statistically significant differences between the estimates of diet diversity indicators in most of the areas studied. For MDD-W, three out of the five datasets had statistically significant differences between reported food intake and what sentinel food lists captured. MDD estimates were significantly different in five out of the six datasets. The magnitude of difference when estimating MDD-W was largest in the Bangladesh ZOI (list-derived estimate was 12 percentage points lower) and smallest in Colombia (4 percentage points lower). MDD differences were largest in the Bangladesh ZOI (list-derived estimate was 8 percentage points lower) and smallest in the Bangladesh ZOR (2 percentage points lower). We also noted the percentage of underestimation by the lists because even where point differences seem small, that difference (e.g. 20 % *v*. 22 % for children in the Bangladesh ZOR) could affect programme implementation and interpretation. Local nutrition programmes often focus on promoting the consumption of certain under-consumed foods and food groups; foods missing from the national sentinel food lists would not be captured and those programmes would not have sufficient information to inform or monitor their work. There is also notable variability in foods missing by country so local knowledge is needed to assess if the sentinel list would be adequate for the programme’s work. In Bangladesh, the sentinel food lists underperformed in that they were unable to capture 90 % of individuals consuming foods from a food group due to missing foods.

Fruit and vegetable food groups had the largest gaps between foods reported in the 24-h dietary recalls and those listed on the sentinel food lists. One reason was because the methodology of all DQQ sentinel food lists excludes onions. As a sentinel food, onions may not be a reliable indicator of vegetable consumption, given their limited nutritional value relative to other foods in that food group^(27)^. That said, the MDD-W vegetable food group does include onions consumed in quantities greater than 15 g. This mainly explains the large discrepancy between the methods in capturing ‘other vegetables’ in Bangladesh, as more than 90% of women reported consuming more than 15 g of onions.

Our findings are similar to a recent study conducted in China, where consumption of sentinel food items from the national-level DQQ sentinel food list accounted for over 90% of respondents (*n* 13 076) who consumed each food group in nearly every province, with some variation by province for the food groups white roots/tubers and vitamin A-rich vegetables^(28)^. Notably, the lists did not perform similarly in the two sub-regions of Bangladesh that we examined.

While differences in our estimates of diet diversity indicators are statistically significant in certain subnational geographic areas, some may not be practically meaningful^(29)^ depending on the intended use of the sentinel food list. In some contexts, the benefits of using rapid, low-burden sentinel food lists that may provide less precise dietary diversity estimates may outweigh the costs (monetary, time, expertise) required to monitor diets using more precise 24-h dietary recalls. For example, national policymakers may not require as much precision on dietary intake as subnational nutrition or agriculture programme managers aiming to show movement in an indicator towards a numeric goal or to increase the consumption of an underutilised nutritious food. However, seemingly small differences could matter to policymakers if the estimated indicator value categorises the subnational geographic area on one side of a threshold, as with Scaling Up Nutrition thresholds (e.g. ^(30))^, with implications for interventions, programmes and budgets.

Other texts have examined the benefits and limitations of different dietary measurement methods^(11,12)^. Hanley-Cook *et al*.^(31)^ found an overestimation of MDD-W by both list-based and open recall methods. Like ours, that study was conducted in sub-regions but, unlike ours, it does not present population-based estimates due to convenience sampling. While they do not provide a direct comparison for this study, the authors also advise consideration of accuracy *v*. simplicity in collecting dietary data for the purpose of making population-level indicator estimates. One particular point that arises from our finding of underestimation of dietary diversity indicators is that if a programme is trying to improve dietary diversity by promoting an underutilised nutritious food that is not on the national sentinel food list and uses the sentinel food list to monitor dietary intake, then the programme’s efforts will not be captured. The food item would need to be added as a separate question apart from the official sentinel list, given that changes to the sentinel lists invalidate them because modifications can result in misclassification of food groups^(17)^. If a sentinel food is missing from the national list, users can report that via www.globaldietquality.org.

Based on the present study, additional sentinel food items, especially for fruit and vegetable food groups, may need to be added to the questionnaire to better capture consumption of those food groups and estimation of MDD-W and MDD at subnational level. In addition, given the differences we observed in estimates of diet diversity, results from national and subnational surveys are likely not comparable if different dietary recall methods are used (i.e. a 24-h open recall *v*. a DQQ sentinel list) to estimate diet diversity indicators. For example, it is common for subnational programmes to compare indicator estimates to national population-based survey estimates. This comparison is not recommended unless the same dietary data collection methods were used. Likewise, a programme should use the same data collection approach at each measurement time point^(31)^ to monitor trends over time.

While we found performance variation in the subnational areas studied, the Bangladesh sentinel food list, in particular, stands out for the differences observed between the reported consumed foods and the sentinel list and therefore may require further adaptations. In circumstances when it is necessary to collect information on additional food items, food groups or diet-related topics, these questions could be added and analysed independently, in alignment with MDD-W and MDD global guidelines^(16,17)^. It may also be advantageous to conduct a rapid assessment of the sentinel food list prior to implementation at the subnational level to assess its performance. A standardised methodology could be developed to test and adapt the sentinel lists in subnational contexts. A more nuanced adaptation of specific food groups, such as the fruit and vegetable food groups, may contribute to improved accuracy of diet diversity indicators at the subnational level. This could be particularly important in subnational areas with relatively high diversity in available foods.

This study has several strengths. We used comprehensive dietary data for women and children from seven ecologically diverse sub-regions of countries around the world to examine our research questions, which will help local nutrition programme implementers plan and monitor their programmes. This study efficiently used existing dietary data, which can be expensive to collect. Our study is timely, as more sentinel food lists are becoming available. Our study provides an inexpensive and robust examination of their utility in subnational areas of countries and programme managers could employ similar methods with subnational dietary data to check list performance before use. Although we observed differences in food groups (e.g. fruits and vegetables) that could be related to seasonality, sentinel lists are developed for use in all seasons; therefore, seasonality should not affect results.

This study also has several limitations. First, we analysed data from specific subnational areas in just six countries across many global regions. Although development of the national sentinel lists follows a similar approach in all countries^(19)^, the qualitative process of interviewing different individuals in each country to create the lists naturally results in some variation in accuracy among countries. Therefore, our findings are not generalisable to other countries or subnational areas and the small number of subnational areas studied within these countries hinders extrapolation. However, the patterns we found can inform decisions about data collection instruments and testing or adaptation that programme implementers may desire.

Although both MDD-W^(16)^ and MDD^(17)^ are validated indicators, they can be influenced by bias in reporting. Dietary recall data are subject to recall bias, and possibly social desirability bias, because they rely on individuals to report consumption during the previous 24 h and respondents may overreport foods they know that they ‘should’ eat^(32)^. In addition, these indicators are qualitative measures so, although MDD-W assumes accurate reporting of consumption of at least 15 g of a food item, that may not have been the quantity consumed. Throughout this analysis, we make the assumption that participants would respond consistently to an open recall and a DQQ (i.e. if they reported a food in an open recall, then they would also respond ‘yes’ to that food if the DQQ were administered to them); some research supports that assumption^(20)^. Further, we used secondary datasets from different sources and had no control over training or data collection and, therefore, data quality may be inconsistent among the datasets. Sample sizes were also variable, although all datasets were representative at the subnational levels indicated. Dishes with multiple ingredients were particularly challenging to classify into food groups because all ingredients and their quantities were often unclear. Thus, those classifications could result in under-reporting foods from mixed dishes that contain ingredients from multiple food groups. In addition, datasets often contained ingredients rather than the final food product (e.g. wheat flour *v*. bread; see online supplementary material, Supplemental Material 3). While we systematically coded all ingredients according to the sentinel food lists, (e.g. coding all flour as grains or whole grains), we do not know how cooks used single ingredients in each instance. Finally, one or more smaller areas may drive observations from subnational areas that combine multiple administrative areas; we did not investigate differences within larger regions.

## Conclusion

This study found that national sentinel food lists, on average, included more than 80 % of foods reportedly consumed by women and children in the assessed datasets, with notable gaps in the fruits and vegetables food groups. We also found that the assessed national sentinel food lists differ from 24-h open dietary recalls in some subnational geographies when measuring the percentage of people who consume a food group and estimating diet diversity indicators (MDD-W and MDD). Programme implementers, policymakers and researchers considering the use of sentinel food lists at subnational level should weigh the benefits against possible inaccuracies and ensure the use of the same data collection method for comparisons over time. Depending on the use and purpose, national sentinel food lists can provide reliable diet quality data at the subnational level for most food groups. However, it is unclear how this may differ by country and sub-region; therefore, it is important to assess the accuracy of the sentinel food lists before using them at the subnational level and to consider adaptations to food groups, if needed, to give programme implementers, policymakers and researchers the level of accurate information that they need about local dietary diversity.

## Supporting information

Vogliano et al. supplementary material Vogliano et al. supplementary material

## Supplementary material

For supplementary material accompanying this paper visit https://doi.org/10.1017/S1368980023002823
