# Supplementary material for: Assessing the performance of national sentinel food lists at subnational levels in six countries
Source: Public Health Nutr. 2023 Dec 15;27(1):e2. doi: 10.1017/S1368980023002823 (PMC10830354; doi:10.1017/S1368980023002823)
Supplement: Vogliano et al. supplementary material [file S1368980023002823sup001.docx]

**Supplementary Materials 1. Classifications**

**MDD-W food groups^*^ matched to Women’s DQQ questionnaire item numbers^†^**

| MDD-W Food Groups | Women’s Diet Quality Questionnaire item numbers |
| --- | --- |
| 1: Grains, white roots and tubers, and plantains | 1: Foods made from grains  2: Whole grains  3: White roots/tubers |
| 2: Pulses (beans, peas, and lentils) | 4: Legumes |
| 3: Nuts and seeds | 21: Nuts and seeds |
| 4: Milk and milk products | 14: Cheese  15: Yogurt  25: Milk from animals |
| 5: Meat, poultry, and fish | 16: Processed meats  17: Unprocessed red meat (ruminants)  18: Unprocessed red meat (non-ruminant)  19: Poultry  20: Fish and seafood |
| 6: Eggs | 13: Eggs |
| 7: Dark green leafy vegetables | 6: Dark green leafy vegetables |
| 8: Other vitamin A-rich fruits and vegetables | 5: Vitamin A-rich orange vegetables  8: Vitamin A-rich fruits |
| 9: Other vegetables | 7: Other vegetables |
| 10: Other fruits | 9: Citrus  10: Other fruits |

*17

**^†^**34

## MDD food groups^*^ matched to Infants and Young Children’s DQQ questionnaire item numbers^†^

| MDD Food Groups | IYC Diet Quality Questionnaire item numbers |
| --- | --- |
| 1: Breast milk^ɪ^ | 4: Breast milk |
| 2: Grains, roots, tubers, and plantains | 7.1: Foods made from grains  7.2: Whole grains  7.3: White roots/tubers |
| 3: Pulses (beans, peas, and lentils); nuts; and seeds | 7.4: Legumes  7.21: Nuts and seeds |
| 4: Dairy products (milk, infant formula, yogurt, cheese) | 6B: Infant formula  6C.25: Milk from animals  7.14: Cheese  7.15: Yogurt |
| 5: Flesh foods (meat, fish, poultry, organ meats) | 7org: Organ meats  7.16: Processed meats  7.17: Unprocessed red meat (ruminants)  7.18: Unprocessed red meat (non-ruminant)  7.19: Poultry  7.20: Fish and seafood |
| 6: Eggs | 7.13: Eggs |
| 7: Vitamin A-rich fruits and vegetables | 7.5: Vitamin A-rich orange vegetables  7.6: Dark green leafy vegetables  7.8: Vitamin A-rich fruits |
| 8: Other fruits and vegetables | 7.7: Other vegetables  7.9: Citrus  7.10: Other fruits |

**^*^**18

**^†^**34

^ɪ^ Though not included as one of the 29 DQQ food groups, breast milk was considered its own food group for children in the calculation of MDD.

**Supplementary Materials 2. Additional Results**

## Table SM 2.1. Percentage of Foods Named in the 24-hour Open Dietary Recalls That Were Not Included in the National-Level Sentinel Food List for the Ten MDD-W Food Groups by Country

| **Food Group** | **Country** | **MDD-W: Percentage of foods named in the 24-hour open dietary recall not included in the national-level sentinel food list** |
| --- | --- | --- |
| Grains, white roots and tubers, and plantains | Kenya | 7.14% |
|  | Malawi | 4.17% |
|  | Colombia | 41.48% |
|  | Bangladesh ZOI | 43.75% |
|  | Bangladesh ZOR | 36.67% |
| Pulses (beans, peas, and lentils) | Kenya | 20.00% |
|  | Malawi | 0.00% |
|  | Colombia | 5.26% |
|  | Bangladesh ZOI | 28.57% |
|  | Bangladesh ZOR | 18.18% |
| Nuts and seeds | Kenya | 0.00% |
|  | Malawi | 12.50% |
|  | Colombia | 30.77% |
|  | Bangladesh ZOI | 100.00% |
|  | Bangladesh ZOR | 100.00% |
| Milk and milk products | Kenya | 0.00% |
|  | Malawi | 0.00% |
|  | Colombia | 2.94% |
|  | Bangladesh ZOI | 14.29% |
|  | Bangladesh ZOR | 0.00% |
| Meat, poultry, and fish | Kenya | 0.00% |
|  | Malawi | 0.00% |
|  | Colombia | 6.72% |
|  | Bangladesh ZOI | 1.35% |
|  | Bangladesh ZOR | 2.90% |
| Eggs | Kenya | 0.00% |
|  | Malawi | 0.00% |
|  | Colombia | 0.00% |
|  | Bangladesh ZOI | 0.00% |
|  | Bangladesh ZOR | 0.00% |
| Dark green leafy vegetables | Kenya | 0.00% |
|  | Malawi | 22.22% |
|  | Colombia | 0.00% |
|  | Bangladesh ZOI | 0.00% |
|  | Bangladesh ZOR | 0.00% |
|  | Kenya | 0.00% |
| Other vitamin A-rich fruits and vegetables | Malawi | 0.00% |
|  | Colombia | 33.33% |
|  | Bangladesh ZOI | 0.00% |
|  | Bangladesh ZOR | 0.00% |
| Other vegetables | Kenya | 33.33% |
|  | Malawi | 30.77% |
|  | Colombia | 19.44% |
|  | Bangladesh ZOI | 46.15% |
|  | Bangladesh ZOR | 40.91% |
| Other fruits | Kenya | 25.00% |
|  | Malawi | 0.00% |
|  | Colombia | 44.44% |
|  | Bangladesh ZOI | 51.85% |
|  | Bangladesh ZOR | 40.91% |
| Overall | Kenya | 10.17% |
|  | Malawi | 6.67% |
|  | Colombia | 21.60% |
|  | Bangladesh ZOI | 21.88% |
|  | Bangladesh ZOR | 17.86% |

**Table SM 2.2. Percentage of Foods Named in the 24-Hour Open Dietary Recalls That Were Not Included in the National-Level Sentinel Food List for the Eight MDD Food Groups by Country**

| **Food Group** | **Country** | **MDD: Percentage of foods named in the 24-hour open dietary recall not included in the national-level sentinel food list** |
| --- | --- | --- |
| Breast milk | Kenya | 0.00% |
|  | Nepal | 0.00% |
|  | Benin | 0.00% |
|  | Colombia | 0.00% |
|  | Bangladesh ZOI | 0.00% |
|  | Bangladesh ZOR | 0.00% |
| Grains, roots, tubers, and plantains | Kenya | 9.09% |
|  | Nepal | 27.27% |
|  | Benin | 5.26% |
|  | Colombia | 43.56% |
|  | Bangladesh ZOI | 45.45% |
|  | Bangladesh ZOR | 39.13% |
| Pulses (beans, peas, and lentils); nuts, and seeds | Kenya | 0.00% |
|  | Nepal | 5.41% |
|  | Benin | 0.00% |
|  | Colombia | 5.71% |
|  | Bangladesh ZOI | 30.00% |
|  | Bangladesh ZOR | 50.00% |
| Dairy products (milk, infant formula, yogurt, cheese) | Kenya | 0.00% |
|  | Nepal | 0.00% |
|  | Benin | 0.00% |
|  | Colombia | 0.00% |
|  | Bangladesh ZOI | 0.00% |
|  | Bangladesh ZOR | 0.00% |
| Flesh foods (meat, fish, poultry, organ meats) | Kenya | 0.00% |
|  | Nepal | 0.00% |
|  | Benin | 0.00% |
|  | Colombia | 3.98% |
|  | Bangladesh ZOI | 0.00% |
|  | Bangladesh ZOR | 3.23% |
| Eggs | Kenya | 0.00% |
|  | Nepal | 0.00% |
|  | Benin | 0.00% |
|  | Colombia | 0.00% |
|  | Bangladesh ZOI | 0.00% |
|  | Bangladesh ZOR | 0.00% |
| Vitamin A-rich fruits and vegetables | Kenya | 0.00% |
|  | Nepal | 16.67% |
|  | Benin | 5.56% |
|  | Colombia | 18.18% |
|  | Bangladesh ZOI | 7.14% |
|  | Bangladesh ZOR | 0.00% |
| Other fruits and vegetables | Kenya | 18.18% |
|  | Nepal | 42.86% |
|  | Benin | 0.00% |
|  | Colombia | 23.40% |
|  | Bangladesh ZOI | 41.67% |
|  | Bangladesh ZOR | 30.00% |
| Overall | Kenya | 6.52% |
|  | Nepal | 17.70% |
|  | Benin | 2.67% |
|  | Colombia | 18.38% |
|  | Bangladesh ZOI | 20.54% |
|  | Bangladesh ZOR | 17.65% |

## Table SM 2.3. Reported Women’s Consumption of Any Qualifying Food and Reported Consumption of at Least One Sentinel Qualifying Food, by Country and MDD-W Food Groups

| **Food Groups** | **Country** | | | | | | | | | |
| --- | --- | --- | --- | --- | --- | --- | --- | --- | --- | --- |
|  | **Kenya** | | **Malawi** | | **Colombia** | | **Bangladesh ZOI** | | **Bangladesh ZOR** | |
|  | **% (n) of respondents who consumed…** | | | | | | | | | |
|  | qualifying foods | sentinel qualifying foods | qualifying foods | sentinel qualifying foods | qualifying foods | sentinel qualifying foods | qualifying foods | sentinel qualifying foods | qualifying foods | sentinel qualifying foods |
| Grains, white roots and tubers, and plantains | 90.69% (497) | 90.69% (497) | 100% (177) | 100% (177) | 99.35% (3,953) | 99.15% (3,945) | 99.95% (1,941) | 99.95% (1,941) | 99.93% (1,444) | 99.86% (1,443) |
| Pulses (beans, peas, and lentils) | 17.33% (95) | 17.15% (94) | 38.98% (69) | 38.98% (69) | 34.83% (1386) | 34.78% (1384) | 40.42% (785) | 37.75% (733) | 19.86% (287) | 18.89% (273) |
| Nuts and seeds | 6.75% (37) | 6.75% (37) | 85.88% (152) | 84.75% (150) | 1.58% (63) | 1.46% (58) | 0.88% (17) | 0% (0) | 2.35% (34) | 0% (0) |
| Milk and milk products | 52.74% (289) | 52.74% (289) | 1.13% (2) | 1.13% (2) | 63.71% (2,535) | 63.66% (2,533) | 8.65% (168) | 8.60% (167) | 5.12% (74) | 5.12% (74) |
| Meat, poultry, and fish | 31.57% (173) | 31.57% (173) | 36.16% (64) | 36.16% (64) | 89.92% (3,578) | 89.45% (3,559) | 79.20% (1,538) | 79.20% (1,538) | 82.49% (1,192) | 82.15% (1,187) |
| Eggs | 5.47% (30) | 5.47% (30) | 2.26% (4) | 2.26% (4) | 54.86% (2,183) | 54.86% (2,183) | 23.48% (456) | 23.48% (456) | 17.92% (259) | 17.92% (259) |
| Dark green leafy vegetables | 61.31% (336) | 61.31% (336) | 71.19% (126) | 68.93% (122) | 1.06% (42) | 1.06% (42) | 31.87% (619) | 31.87% (619) | 25.33% (366) | 25.33% (366) |
| Other vitamin A -rich fruits and vegetables | 10.95% (60) | 10.95% (60) | 19.21% (34) | 19.21% (34) | 42.02% (1672) | 36.69% (1460) | 12.10% (235) | 12.10% (235) | 5.68% (82) | 5.68% (82) |
| Other vegetables | 53.47% (293) | 53.29% (292) | 62.15% (110) | 62.15% (110) | 52.80% (2,101) | 50.29% (2,001) | 97.99% (1,903) | 60.81% (1,181) | 99.52% (1,438) | 83.32% (1,204) |
| Other fruits | 18.80% (103) | 18.43% (101) | 10.17% (18) | 10.17% (18) | 35.89% (1,428) | 31.39% (1,249) | 26.78% (520) | 24.00% (466) | 26.37% (381) | 25.33% (366) |

## Table SM 2.4. Reported Children’s Consumption of Any Qualifying Food and Reported Consumption of at Least One Sentinel Qualifying Food, by Country and MDD Food Groups

| **Food Groups** | **Country** | | | | | | | | | | | |
| --- | --- | --- | --- | --- | --- | --- | --- | --- | --- | --- | --- | --- |
|  | **Kenya** | | **Nepal** | | **Benin** | | **Colombia** | | **Bangladesh ZOI** | | **Bangladesh ZOR** | |
|  | **% (n) of respondents who consumed…** | | | | | | | | | | | |
|  | qualifying foods | sentinel qualifying foods | qualifying foods | sentinel qualifying foods | qualifying foods | sentinel qualifying foods | qualifying foods | sentinel qualifying foods | qualifying foods | sentinel qualifying foods | qualifying foods | sentinel qualifying foods |
| Breast milk | 80.48% (235) | 80.48% (235) | 94.09% (701) | 94.09% (701) | 90.40% (1,130) | 90.40% (1,130) | 52.25% (813) | 52.25% (813) | 81.32% (309) | 81.32% (309) | 68.49% (250) | 68.49% (250) |
| Grains, roots, tubers, and plantains | 97.95% (286) | 97.95% (286) | 99.20% (739) | 98.52% (734) | 96.40% (1,205) | 96.40% (1,205) | 97.04% (1,510) | 94.02% (1,463) | 64.74% (246) | 59.21% (225) | 67.12% (245) | 66.03% (241) |
| Pulses (beans, peas, and lentils); nuts; and seeds | 11.30% (33) | 11.30% (33) | 88.19% (657) | 87.79% (654) | 24.32% (304) | 24.32% (304) | 36.12% (562) | 35.99% (560) | 11.58% (44) | 11.05% (42) | 12.06% (44) | 11.78% (43) |
| Dairy products (milk, infant formula, yogurt, cheese) | 42.81% (125) | 42.81% (125) | 78.39% (584) | 78.39% (584) | 1.44% (18) | 1.44% (18) | 83.10% (1,293) | 83.10% (1,293) | 14.47% (55) | 14.47% (55) | 4.93% (18) | 4.93% (18) |
| Flesh foods (meat, fish, poultry, organ meats) | 32.88% (96) | 32.88% (96) | 23.76% (177) | 23.77% (177) | 71.48% (898) | 71.48% (898) | 76.16% (1,185) | 76.09% (1,184) | 28.16% (107) | 28.16% (107) | 27.67% (101) | 27.40% (100) |
| Eggs | 7.88% (23) | 7.88% (23) | 22.15% (165) | 22.15% (165) | 1.92%  (24) | 1.92%  (24) | 52.96% (824) | 52.96% (824) | 20.00% (76) | 20.00% (76) | 20.55% (75) | 20.55% (75) |
| Vitamin A–rich fruits and vegetables | 48.29% (141) | 48.29% (141) | 33.02% (246) | 29.93% (223) | 72.24% (903) | 64.16% (802) | 63.11% (982) | 58.93% (917) | 12.11% (46) | 12.11% (46) | 9.86% (36) | 9.86% (36) |
| Other fruits and vegetables | 66.43% (194) | 66.43% (194) | 81.75% (609) | 76.11% (567) | 74.32% (929) | 74.32% (929) | 76.09% (1,184) | 71.85% (1,118) | 42.11% (160) | 23.16% (88) | 41.92% (153) | 20.82% (76) |

**Table SM 2.5. MDD-W Among Women 15–49 Years Old by Reported Consumption of Any Qualifying MDD-W Foods and Any Sentinel Qualifying Foods**

| **Kenya** | | **Malawi** | | **Colombia*** | | **Bangladesh ZOI*** | | **Bangladesh ZOR*** | |
| --- | --- | --- | --- | --- | --- | --- | --- | --- | --- |
| **% (95% confidence interval [CI]) of respondents who achieved MDD-W with consumption of…** | | | | | | | | | |
| any qualifying foods | any sentinel qualifying foods | any qualifying foods | any sentinel qualifying foods | any qualifying foods | any sentinel qualifying foods | any qualifying foods | any sentinel qualifying foods | any qualifying foods | any sentinel qualifying foods |
| 26.50%  (22.78%,30.15%) | 26.30%  (22.59%,29.96%) | 39.60% (32.34%,46.75%) | 39.00% (31.80%,46.17%) | 56.30% (54.70%,57.79%) | 52.60% (51.05%,54.15%) | 36.60% (34.42%,38.70%) | 25.10% (23.45%,27.00%) | 24.30% (22.08%,26.50%) | 19.80% (17.73%,21.84%) |

* Statistically significant at *p*<0.05

**Table SM 2.6. MDD among Children 6–23 Months by Reported Consumption of Any Qualifying MDD Food and Any Sentinel Qualifying Foods**

| **Kenya** | | **Nepal** | | **Benin** | | **Colombia** | | **Bangladesh ZOI** | | **Bangladesh ZOR** | |
| --- | --- | --- | --- | --- | --- | --- | --- | --- | --- | --- | --- |
| **% (95% CI) of respondents who achieved MDD with consumption of…** | | | | | | | | | | | |
| any qualifying foods | any sentinel qualifying foods | any qualifying foods | any sentinel qualifying foods | any qualifying foods | any sentinel qualifying foods | any qualifying foods | any sentinel qualifying foods | any qualifying foods | any sentinel qualifying foods | any qualifying foods | any sentinel qualifying foods |
| 33.90% (28.50%, 39.30%) | 33.90% (28.50%, 39.30%) | 73.80% (70.70%, 77.00%) | 71.00% (67.80%, 74.30%) | 61.80% (59.20%, 64.50%) | 56.70% (54.00%, 59.50%) | 74.30% (72.10%, 76.50%) | 71.50% (69.30%, 73.80%) | 19.70% (15.70%, 23.70%) | 11.60% (8.40%, 14.80%) | 8.50% (5.60%, 11.40%) | 6.60% (4.00%, 9.10%) |

**Supplementary Materials 3. Consumption of food items not included in national sentinel food lists**

**Table SM 3.1. Food Items Listed in the 24-hour Dietary Recalls That Were Not Included in the National Sentinel Food Lists by Country and MDD-W/MDD Food Groups**

| **Country** | | | | | | | |
| --- | --- | --- | --- | --- | --- | --- | --- |
| **Food Groups** | Kenya | Malawi | Nepal | Benin | Colombia | Bangladesh ZOI | Bangladesh ZOR |
| **MDD-W** | | | | | | | |
| Grains, white roots and tubers, and plantains | Whole meal maize | Chingoe maize, white |  |  | Processed crackers of various flavors  Oats/oatmeal  Whole wheat flour  Pizza dough  Breakfast cereals of various flavors  Corn  Pancake mix  Cornmeal/flour  Cuchuco (soup made up of corn, barley/wheat, and beans)  Whole wheat dough  Puff pastry  Cornmeal dough  Granola  Flour tortillas  Barley  Plantain flour  Turnip  Granola bar  Potato flour  Taro  Rice flour  Red Achira flour  Infant cereal  Yacon  Dumpling dough  Corn tortillas  Wheat flour | White Atta (wheat flour)  Rice flour  Rice bran  Taro of various species  Maida (refined wheat flour)  Elephant foot yam  Toast  Green banana  Turnip  LAL Atta (Wheat flour)  Suji (cream of wheat)  Whole wheat | White Atta (wheat flour)  Rice flour  Rice bran  Taro  Maida (refined wheat flour)  Elephant foot yam  Toast  Green banana  Turnip  LAL Atta (Wheat flour)  Suji (cream of wheat) |
| Pulses (beans, peas, and lentils) | Soybean meat substitute |  |  |  | Soy milk | Sheem (bean)  Bean seed/flat bean  Seed  Soybean  Daler Bori | Sheem (bean)  Bean seed/flat bean  Seed  Soybean |
| Nuts and seeds |  | Bambara nuts |  |  | Cashew, almond, Brazil nut, flaxseed | Badam  Almonds  Any other seed | Badam  Any other seed |
| Milk and milk products |  |  |  |  | Prenatal and postnatal nutritional shake (mom Similac), bechamel sauce, whey | Biomil infant formula |  |
| Meat, poultry, and fish |  |  |  |  | Bacon, goose, turkey, rabbit, pork liver, chicken neck, duck, goat, lamb, lowland paca (large rodent species), quail, pork kidney, pork small intestines | Panch Mishali  Buffalo | Buffalo  Pork  Panch Mishali |
| Eggs |  |  |  |  |  |  |  |
| Dark green leafy vegetables |  | Okra leaves (various cooking methods) |  |  |  |  |  |
| Other vitamin A-rich fruits and vegetables |  |  |  |  | Tree tomato (tamarillo), banana passionfruit, lulo, Peruvian groundcherry, passionfruit |  |  |
| Other vegetables | Onion, fresh peas | Onion, fresh pea, red onion |  |  | Onion, asparagus, yellow zucchini, chayote, bean sprouts, radish, squash | Onion  Onion/garlic stalk  Plantain flower  Pea  Lottori  Snake gourd  Ridge gourd  Amilla gulo  Sponge gourd  Drumstick | Onion  Plantain flower  Bamboo shoot  Onion/garlic stalk  Pea  Snake gourd  Amilla gulo |
| Other fruits | Lemon, jackfruit |  |  |  | Blackberry, kiwi, mangosteen, plum, green mango, mamoncillo, cherry, watermelon, feijoa, yellow dragon fruit, maduro, pulpa  agraz berry, araza, borojo, star fruit, tamarind, rose-apple, guama, coconut, wild sweetsop, cashew fruit/cashew apple,  dried fruit | Tamarind  Elephant apple  Pomegranate  Cucumber  Grapes  Java apple  Dates  Lemon  Emblic  Sapodilla/sapota  Wood apple  Other fruit  Asian pears | Tamarind  Cucumber  Wood apple  Pomegranate  Grapes  Dates  Lemon  Emblic  Palm |
| **MDD** | | | | | | | |
| Breast milk |  |  |  |  |  |  |  |
| Grains, roots, tubers, and plantains | Whole meal maize |  | Oats, Popcorn  Muesli Weetabix Infant cereals Cereal Vermicelli Semolina Cornflakes Fusilli pasta Nimki  Taro | Amaranthus cruentus | Processed crackers of various flavors  Oats/oatmeal  Infant cereal  Rice flour  Corn  Wheat flour  Papilla Nestum, crema de arroz  Cornmeal/flour  Plantain flour  Barley  Cuchuco (soup made up of corn, barley/wheat, and beans)  Pizza dough  Yuca starch  Flour tortilla  Pancake mix  Granola bar  Wheat dough  Whole wheat flour  Taro  Yuca flour  Red achira flour  Cornmeal dough  Puff pastry | Green banana  Turnip  Toast  Suji (cream of wheat/barley)  Maida (wheat flour without bran)  Rice flour  White Atta (wheat flour)  Rice bran  Taro  Sagu/sago | White Atta (wheat flour)  Toast  Maida (wheat flour without bran)  Suji (cream of wheat/barley)  Rice bran  Rice flour  LAL Atta (Wheat flour)  Sagu/sago |
| Pulses (beans, peas, and lentils); nuts and seeds |  |  | Dried pea Masaura |  | Soy milk | Sheem (bean)  Soybean  Bean seed/flat bean  Seed  Almonds  Any other seed | Sheem (bean)  Bean seed  Flat bean seed  Badam  Any other seed |
| Dairy products (milk, infant formula, yogurt, cheese) |  |  |  |  |  |  |  |
| Flesh foods (meat, fish, poultry, organ meats) |  |  |  |  | Bacon, goose, chicken neck, turkey, quail, lowland paca (large rodent species) |  | Pork  Panch Mishali |
| Eggs |  |  |  |  |  |  |  |
| Vitamin A–rich fruits and vegetables |  |  | Green onion, lamb's quarter leaves | Red pepper | Tree tomato (tamarillo), lulo, passionfruit, banana passionfruit |  |  |
| Other fruits and vegetables | Fresh onion, onion tuber |  | Strawberry, dried coconut, dried dates, fresh coconut, red onion, button mushroom, fresh pea, French bean, oyster mushroom, maize, cucumber, capsicum, asparagus, bamboo shoot, barela |  | Plum, onion, watermelon, blackberry, leeks, kiwi, agraz berry, coconut, sweet pepper, mangosteen, yellow zucchini, borojo, rose-apple, tamarind, green mango, bean sprouts, star fruit, chayote, squash, cashew fruit/cashew apple | Onion  Pomegranate  Grapes  Tamarind  Sapodilla/sapota  Dates  Other fruit  Cucumber | Onion  Grapes  Pomegranate |

**Table SM 3.2. Percentage of Women and Children Who Reported Consuming Specific Food Items not Included in the National Level Sentinel Food Lists for Food Groups that Consistently had Higher Percentages of Foods Not Captured, by Country**

| **Food Groups** | **Kenya** | | **Malawi** | | **Nepal** | | **Benin** | | **Colombia** | | **Bangladesh ZOI** | | **Bangladesh ZOR** | |
| --- | --- | --- | --- | --- | --- | --- | --- | --- | --- | --- | --- | --- | --- | --- |
|  | **Food Item** | **Percentage of respondents who reported consuming the food item** | **Food Item** | **Percentage of respondents who reported consuming the food item** | **Food Item** | **Percentage of respondents who reported consuming the food item** | **Food Item** | **Percentage of respondents who reported consuming the food item** | **Food Item** | **Percentage of respondents who reported consuming the food item** | **Food Item** | **Percentage of respondents who reported consuming the food item** | **Food Item** | **Percentage of respondents who reported consuming the food item** |
| **MDD-W** | | | | | | | | | | | | | |  |
| Grains, roots, tubers, and plantains | Whole meal maize | 0.18% | Chingowe, maize | 2.82% |  |  |  |  | Cornmeal/ flour | 11.68% | Rice flour | 1.60% | Rice flour | 1.87% |
|  |  |  |  |  |  |  |  |  | Oats/ Oatmeal | 3.04% | Suji (Cream of wheat) | 0.21% | Suji (Cream of wheat) | 0.28% |
|  |  |  |  |  |  |  |  |  | Bore | 0.13% | Whole wheat | 0.57% | LAL Atta | 0.07% |
|  |  |  |  |  |  |  |  |  | Turnip | 0.08% | LAL Atta | 1.80% | White Atta | 3.53% |
|  |  |  |  |  |  |  |  |  | Barley | 0.33% | White Atta | 12.41% | Maida | 2.04% |
|  |  |  |  |  |  |  |  |  | Cuchuco (soup made up of corn, barley/ wheat, and beans) | 0.53% | Rice bran | 2.78% | Rice bran | 0.21% |
|  |  |  |  |  |  |  |  |  | Processed crackers of various types | 16.16% | Green banana | 0.46% | Green banana | 0.97% |
|  |  |  |  |  |  |  |  |  | Rice flour | 0.03% | Other item | 3.81% | Taro | 1.04% |
|  |  |  |  |  |  |  |  |  | Wheat flour | 6.60% | Taro | 2.42% | Elephant foot yam | 0.07% |
|  |  |  |  |  |  |  |  |  | Corn | 5.02% | Elephant Foot yam | 0.46% | Turnip | 0.07% |
|  |  |  |  |  |  |  |  |  | Cornmeal dough | 1.86% | Turnip | 3.04% | Toast | 0.76% |
|  |  |  |  |  |  |  |  |  | Taro | 0.05% | Toast | 4.99% |  |  |
|  |  |  |  |  |  |  |  |  | Yuca flour | 0.10% |  |  |  |  |
|  |  |  |  |  |  |  |  |  | Red achira flour | 0.05% |  |  |  |  |
|  |  |  |  |  |  |  |  |  | Potato flour | 0.03% |  |  |  |  |
|  |  |  |  |  |  |  |  |  | Pancake mix | 0.05% |  |  |  |  |
|  |  |  |  |  |  |  |  |  | Granola bar | 0.13% |  |  |  |  |
|  |  |  |  |  |  |  |  |  | Breakfast cereal | 0.50% |  |  |  |  |
|  |  |  |  |  |  |  |  |  | Granola | 0.65% |  |  |  |  |
|  |  |  |  |  |  |  |  |  | Puff pastry | 0.18% |  |  |  |  |
|  |  |  |  |  |  |  |  |  | Flour tortilla | 0.70% |  |  |  |  |
|  |  |  |  |  |  |  |  |  | Corn tortilla | 0.05% |  |  |  |  |
|  |  |  |  |  |  |  |  |  | Pizza dough | 1.99% |  |  |  |  |
|  |  |  |  |  |  |  |  |  | Plantain flour | 0.03% |  |  |  |  |
|  |  |  |  |  |  |  |  |  | Yacon | 0.03% |  |  |  |  |
|  |  |  |  |  |  |  |  |  | Dumpling dough | 0.03% |  |  |  |  |
| Other vegetables | Onions | 0.55% | Onion | 5.65% |  |  |  |  | Onion | 21.26% | Flat bean seed | 0.62% | Bamboo shoot | 0.07% |
|  | Fresh green peas | 0.36% |  |  |  |  |  |  | Asparagus | 0.03% | Sheem bean | 23.43% | Flat bean seed | 12.25% |
|  |  |  |  |  |  |  |  |  | Chayote | 0.28% | Cucumber | 0.57% | Sheem bean | 29.07% |
|  |  |  |  |  |  |  |  |  | Radish | 0.05% | Sponge gourd | 0.05% | Cucumber | 1.94% |
|  |  |  |  |  |  |  |  |  | Squash | 0.03% | Ribbed gourd | 0.05% | Snake Gourd | 0.07% |
|  |  |  |  |  |  |  |  |  | Bean sprouts | 0.08% | Drum stick | 0.05% | Kolar Mocha | 0.35% |
|  |  |  |  |  |  |  |  |  | Yellow zucchini | 0.03% | Snake gourd | 0.05% | Onion/garlic stalk | 0.62% |
|  |  |  |  |  |  |  |  |  |  |  | Kolar Mocha | 0.72% | Panch Mishali | 1.52% |
|  |  |  |  |  |  |  |  |  |  |  | Onion/garlic stalk | 1.69% | Onion | 98.13% |
|  |  |  |  |  |  |  |  |  |  |  | Panch Mishali | 0.77% |  |  |
|  |  |  |  |  |  |  |  |  |  |  | Onion | 94.38% |  |  |
|  |  |  |  |  |  |  |  |  |  |  | Other item | 0.05% |  |  |
| Other fruits | Jackfruit | 0.18% |  |  |  |  |  |  | Wild sweetsop | 0.03% | Amilla gulo | 0.05% | Amilla gulo | 0.21% |
|  | Lemon | 0.18% |  |  |  |  |  |  | Araza | 0.15% | Other fruit | 0.10% | Wood apple | 0.07% |
|  |  |  |  |  |  |  |  |  | Borojo | 0.23% | Wood apple | 0.10% | Grapes | 0.90% |
|  |  |  |  |  |  |  |  |  | Star fruit | 0.08% | Grapes | 2.94% | Tamarind | 0.90% |
|  |  |  |  |  |  |  |  |  | Cherry | 0.08% | Tamarind | 0.72% | Pomegranate | 0.35% |
|  |  |  |  |  |  |  |  |  | Plum | 0.33% | Pomegranate | 0.82% | Lemon | 0.14% |
|  |  |  |  |  |  |  |  |  | Coconut | 0.43% | Lemon | 0.31% | Dates | 0.07% |
|  |  |  |  |  |  |  |  |  | Feijoa | 0.05% | Dates | 0.82% | Palm | 0.14% |
|  |  |  |  |  |  |  |  |  | Guama | 0.05% | Chalta | 0.36% | Emblic | 0.07% |
|  |  |  |  |  |  |  |  |  | Kiwi | 0.35% | Sapodila | 0.31% |  |  |
|  |  |  |  |  |  |  |  |  | Mamoncillo (Spanish lime) | 0.08% | Elephant apple | 0.26% |  |  |
|  |  |  |  |  |  |  |  |  | Mangosteen | 0.08% | Java apple | 0.05% |  |  |
|  |  |  |  |  |  |  |  |  | Blackberry | 4.00% | Asian pears | 0.05% |  |  |
|  |  |  |  |  |  |  |  |  | Watermelon | 0.73% | Emblic | 0.05% |  |  |
|  |  |  |  |  |  |  |  |  | Yellow dragonfruit | 0.15% |  |  |  |  |
|  |  |  |  |  |  |  |  |  | Cashew fruit/ cashew apple | 0.05% |  |  |  |  |
|  |  |  |  |  |  |  |  |  | Rose apple | 0.08% |  |  |  |  |
|  |  |  |  |  |  |  |  |  | Tamarind | 0.05% |  |  |  |  |
|  |  |  |  |  |  |  |  |  | Agraz berry | 0.05% |  |  |  |  |
|  |  |  |  |  |  |  |  |  | Green mango | 1.18% |  |  |  |  |
|  |  |  |  |  |  |  |  |  | Dried fruit | 0.03% |  |  |  |  |
| **MDD** | | | | | | | | | | | | | |  |
| Grains, roots, tubers, and plantains | Wholemeal maize | 0.34% |  |  | Infant Cereal | 8.18% | Amaranthus cruentus | 0.32% | Cornmeal/ flour | 6.93% | Rice Flour | 1.84% | Rice flour | 0.27% |
|  |  |  |  |  | Cereal | 1.07% |  |  | Oats/ Oatmeal | 15.52% | Cream of wheat | 12.37% | Suji (Cream of wheat) | 3.29% |
|  |  |  |  |  | Muesli | 0.13% |  |  | Bore | 0.19% | White Atta | 2.89% | Whole wheat | 0.27% |
|  |  |  |  |  | Nimki | 0.13% |  |  | Corn | 3.08% | Maida | 1.05% | LAL Atta | 0.27% |
|  |  |  |  |  | Oats | 1.34% |  |  | Barley | 1.09% | Sagu | 1.05% | White Atta | 1.64% |
|  |  |  |  |  | Fusili pasta | 0.27% |  |  | Cuchuco (soup made up of corn, barley/ wheat, and beans) | 1.02% | Rice bran | 1.32% | Maida | 0.55% |
|  |  |  |  |  | Popcorn | 2.00% |  |  | Processed crackers of various types | 22.99% | Green banana | 1.05% | Sagu | 1.10% |
|  |  |  |  |  | Semolina | 1.88% |  |  | Rice flour | 0.19% | Taro | 0.26% | Rice bran | 0.27% |
|  |  |  |  |  |  |  |  |  | Wheat flour | 5.00% | Turnip | 1.05% | Toast | 0.82% |
|  |  |  |  |  | Taro | 0.27% |  |  | Cornmeal dough | 0.13% | Toast | 2.37% |  |  |
|  |  |  |  |  | Weetabix | 0.13% |  |  | Yuca flour | 1.22% |  |  |  |  |
|  |  |  |  |  |  |  |  |  | Red Achira flour | 0.06% |  |  |  |  |
|  |  |  |  |  |  |  |  |  | Pancake mix | 0.06% |  |  |  |  |
|  |  |  |  |  |  |  |  |  | Granola bar | 0.06% |  |  |  |  |
|  |  |  |  |  |  |  |  |  | Breakfast cereal | 0.06% |  |  |  |  |
|  |  |  |  |  |  |  |  |  | Granola | 0.06% |  |  |  |  |
|  |  |  |  |  |  |  |  |  | Puff pastry | 0.06% |  |  |  |  |
|  |  |  |  |  |  |  |  |  | Flour tortilla | 0.19% |  |  |  |  |
|  |  |  |  |  |  |  |  |  | Pizza dough | 0.32% |  |  |  |  |
|  |  |  |  |  |  |  |  |  | Infant cereal | 16.89% |  |  |  |  |
|  |  |  |  |  |  |  |  |  | Plantain flour | 0.90% |  |  |  |  |
| Other fruits and vegetables | Fresh onion | 61.30% |  |  | Asparagus | 0.13% | Red pepper | 68.64% | Onion | 26.54% | Other fruit | 0.26% | Flat bean seed | 1.37% |
|  | Onion tuber | 0.34% |  |  | Bamboo shoot | 0.13% |  |  | Chayote | 0.13% | Flat bean seed | 0.79% | Sheem Bean | 5.21% |
|  |  |  |  |  | Barela | 0.27% |  |  | Borojo | 0.45% | Sheem bean | 2.37% | Grapes | 1.37% |
|  |  |  |  |  | Capsicum | 0.54% |  |  | Star fruit | 0.13% | Cucumber | 0.26% | Pomegranate | 0.27% |
|  |  |  |  |  | Dried coconut | 2.42% |  |  | Plum | 0.45% | Grapes | 8.95% | Oanch Mishali | 0.27% |
|  |  |  |  |  | Fresh coconut | 0.13% |  |  | Coconut | 0.58% | Tamarind | 1.05% | Onion | 30.96% |
|  |  |  |  |  | Cucumber | 0.27% |  |  | Kiwi | 0.32% | Pomegranate | 3.42% |  |  |
|  |  |  |  |  | Dates | 4.83% |  |  | Mangosteen | 0.06% | Dates | 0.26% |  |  |
|  |  |  |  |  | French bean | 3.09% |  |  | Cashew fruit/cashew apple | 0.06% | Sapodila | 0.53% |  |  |
|  |  |  |  |  | Maize | 0.94% |  |  | Blackberry | 6.04% | Onion | 29.74% |  |  |
|  |  |  |  |  | Button mushroom | 0.54% |  |  | Watermelon | 0.77% |  |  |  |  |
|  |  |  |  |  | Oyster mushroom | 1.61% |  |  | Rose apple | 0.06% |  |  |  |  |
|  |  |  |  |  | Onion | 40.81% |  |  | Tamarind | 0.06% |  |  |  |  |
|  |  |  |  |  | Fresh peas | 3.89% |  |  | Sweet pepper | 0.19% |  |  |  |  |
|  |  |  |  |  | Strawberry | 0.40% |  |  | Squash | 0.06% |  |  |  |  |
|  |  |  |  |  |  |  |  |  | Bean sprouts | 0.13% |  |  |  |  |
|  |  |  |  |  |  |  |  |  | Yellow zucchini | 0.06% |  |  |  |  |
|  |  |  |  |  |  |  |  |  | Agraz | 0.13% |  |  |  |  |
|  |  |  |  |  |  |  |  |  | Green mango | 0.06% |  |  |  |  |
